# The role of mGlu4 receptors within the nucleus accumbens in acquisition and expression of morphine-induced conditioned place preference in male rats

**DOI:** 10.1186/s12868-021-00627-2

**Published:** 2021-03-21

**Authors:** Zahra Ebrahimi, Nazanin Kahvandi, Alireza Komaki, Seyed Asaad Karimi, Marzieh Naderishahab, Abdolrahman Sarihi

**Affiliations:** 1 Neurophysiology Research Center, Hamadan University of Medical Sciences, Hamadan, Iran; 2 Department of Neuroscience, School of Sciences and Advanced Technology in Medicine, Hamadan University of Medical Sciences, Hamadan, Iran; 3 Department of Physiology, Faculty of Medicine, Hamadan University of Medical Sciences, Hamadan, Iran

**Keywords:** Metabotropic glutamate receptor type 4, Nucleus accumbens, Conditioned place preference, Morphine, Rat

## Abstract

**Background:**

Several studies have shown that glutamate neurotransmission in the nucleus accumbens (NAc) is required for the development of morphine-induced conditional place preference (CPP). In addition, metabotropic glutamate receptors (mGluRs) in NAc play important roles in the reward pathways. However, the precise role of mGluR4 in different steps of the morphine-induced CPP is less well known. In the present study the effect of bilateral intra-accumbal infusion of VU0155041, as a specific mGluR4 agonist on the acquisition and expression of morphine induced CPP in male Wistar rats was investigated. The animals were bilaterally implanted with guide cannulae above the NAc. In the first step of the study, the VU0155041 was administered at doses of 10, 30 and 50 μg/0.5 μL saline per side into the NAc during the 3 days of morphine (5 mg/kg) conditioning (acquisition) phase of morphine-induced CPP. In the second step of the study, the rats bilaterally received VU0155041 at the dose of 50 μg/0.5 μL, 5 min before the post-conditioning test in order to check the effect of VU0155041 on the expression of morphine-induced CPP.

**Results:**

The results showed that the intra-accumbal injection of VU0155041 inhibits the acquisition of morphine-induced CPP in a dose dependent manner, but had no effect on expression.

**Conclusions:**

The data indicated that intra-NAc administration of VU0155041 dose dependently blocks the establishment of morphine-induced CPP and reduces the rewarding properties of morphine. These effects may be related to changes in glutamate activity in the NAC and/or learning dependent mechanism of glutamate neurotransmission in reward pathway(s).

## Background

Drug craving and seeking behavior is a characteristics of drug abuse after prolonged abstinence [1]. The attraction and motivational property of a stimulus is called reward that induces appetitive behavior and usually creates a conscious experience of pleasure [2]. Alcohol, nicotine, cocaine, morphine, and heroin have rewarding effects which play a chief role in the initiation and maintenance of the drug-taking habit [3]. Reward circuits especially the mesolimbic reward pathway links the ventral tegmental area (VTA) of the midbrain via the medial forebrain bundle to the nucleus accumbens (NAc) of the striatum [4]. The NAc is one of the most important neural elements in the reward pathway which is located inside the ventral striatum [5, 6].

Previous studies have shown that dopamine, glutamate and GABA are involved in the reward circuit, while dopamine and glutamate are the most important neurotransmitters of these pathways [7, 8]. Anatomical and electrophysiological studies have shed light on the fact that glutamatergic input into the NAc originating from the medial prefrontal cortex, VTA, and amygdala play an important role in addictive behaviors [8, 9].

Glutamate inputs into the NAc are involved in conditioned place preference (CPP) induced by morphine, cocaine, or amphetamine [10]. Withdrawal from chronic drug exposure causes a number of alterations in glutamatergic transmission within the NAc [10]. Glutamatergic inputs into the NAc originate from the prefrontal cortex, thalamus, basolateral amygdala (BLA), and hippocampus [11–13]. The NAc integrates information from cortical and limbic structures to mediate goal-directed behaviors and is a major input structure of the basal ganglia [10]. Disrupted synaptic plasticity in the NAc following chronic exposure to several classes of drugs of abuse, allows drug-associated cues to engender a pathologic motivation for drug seeking [10].

Glutamate transmission is mediated by ionotropic (iGluRs) and metabotropic glutamate receptors (mGluRs) [14–16]. Metabotropic glutamate receptors have eight subtypes and are classified into three groups including: group I (mGluR1 and mGluR5), group II (mGluR2 and mGluR3), and group III (mGluR4, mGluR6, mGluR7, and mGluR8) depending on their signal transduction pathways, sequence homology, and pharmacological selectivity. mGluRs are G-protein coupled receptors and group III of mGluRs are coupled with the Gi/o proteins [17].

The NAc expresses high density of mGluRs, especially mGlu4, 5 and 8 receptors. Previous studies have reported that glutamate transmission into the NAc plays an important role in different phase of opioid rewards including: expression (7), extinction [18, 19], and reinstatement [18–20] of morphine-induced CPP. However, the precise role of mGluR4 in morphine-induced CPP is unclear.

In our previous reports we identified the role mGluR2/3, mGluR5, mGluR7 and mGluR8 in NAc on acquisition and expression of morphine-induced CPP in rats [7, 21–23]. CPP model was used to measure the motivational effects of objects or experiences and is a form of Pavlovian conditioning [24]. In this task by measuring the amount of time which a subject spends in an area that has been associated with a stimulus, researchers can infer the liking behavior of the animal for the stimulus [25]. CPP as a behavioural model has been developed to study the effects of drugs and non-drug treatments on motivation in experimental animals [26, 27]. At the present, CPP is widely used to test context associations based on the rewarding properties of an unconditioned stimulus in many subjects including rodents [28], flies [29], C. elegans [30, 31], planaria [32, 33], primates [34] and humans [25, 35, 36].

mGluRs have been identified as potential targets for the treatment of drug addiction. It has been suggested the type specificity and phase dependency for the role of mGluRs in morphine induced-CPP. Previously, it has been reported that microinjection of the LY379268 (as a mGluR2/3 agonist) into the NAc inhibits the acquisition and expression (7), and attenuates extinction latencies and the reinstatement of morphine-induced CPP in rats [37]. Moreover, it has been reported that injection of mGluR5 antagonist into the NAc reduces rewarding properties of morphine [22]. In addition, it was shown that intra-accumbal injection of AMN082 (mGluR7 allosteric agonist) inhibits the acquisition of morphine-induced CPP in rats [21]. Also, it has been shown that mGluR7 orthosteric agonist, LSP2-9166, can block morphine CPP expression and reinstatement after extinction [38]. It is indicated that activation of mGluR4 has an important effect on the rewarding properties of alcohol [39] and recently Zaniewska et al. showed that mGluR4 activation reduces cocaine- but not nicotine-induced locomotor sensitization [40]. mGluR4 receptors are widely distributed in different parts of the brain including the cerebellar cortex, globus pallidus and ventral pallidum (VP), olfactory tubercle, striatum, entopeduncular nucleus, the sensory relay nuclei of the thalamus, neocortex, piriform cortex, hippocampus, lateral and basolateral amygdaloid nuclei, and in the superficial grey of the superior colliculus [41, 42]. Specifically, the mGluR4 has high density in brain regions involved in reward circuits such as NAc, VP, and VTA [41, 43–45]. This anatomical distribution of mGluR4 suggests that this receptor may play a critical role in drug dependence. The mGluR4 is primarily localized presynaptically in GABAergic and glutamatergic terminals and is involved in the regulation of glutamate and GABA release [41, 45] and acts as inhibitory presynaptic receptor and reduces synaptic transmission [46].

Taken together, although the precise role of mGluR4 in morphine-induced CPP is unclear but it seems that there is a type specificity in the role of mGluRs in different phase of drug abuse. Therefore, the goal of the current study was to assess the involvement of intra-accumbal mGluR4 in the acquisition and expression of morphine-induced CPP in male rats.

## Results

### Effects of intra-accumbal microinjection of mGluR4 receptor agonist, VU0155041, on the acquisition of morphine-induced CPP

**Fig. 1:**
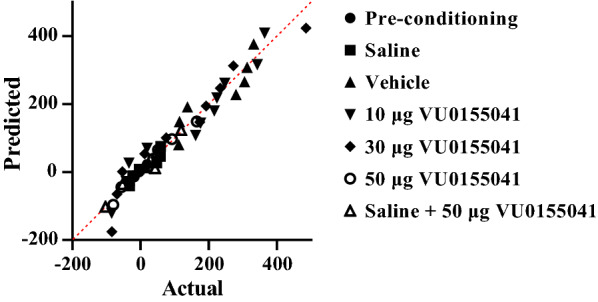
Q-Q plot for distribution of conditioning score data in the conditioning days. These data did not pass normality test**.** The data normality test was performed using Shapiro–Wilk test

To investigate the effects of mGluR4 agonist on the acquisition of morphine-induced CPP, intra-accumbal injection of VU0155041 (10, 30 and 50 μg/0.5 μL) was bilaterally done 5 min prior to each morphine injection during the 3-day conditioning phase (Fig. 1). In this study, the saline group animals received subcutaneous injection of saline (as a solvent of morphine) instead of morphine during the conditioning phase. The vehicle group animals received subcutaneous injection of morphine (5 mg/kg) during the conditioning phase along with intra-NAc microinjection of saline (as a solvent of VU0155041). In the present study, rats with misplaced cannula were considered as an anatomical control group [21, 47]. The findings revealed that there is no significant difference between the conditioning score (CS) in the anatomical control (223.6 ± 17.6) and vehicle group (227.5 ± 35) (data not shown in graph). This means that application of VU0155041 in the areas surrounding the NAc had no effect on conditioning scores and the observed results are most likely due to the effect of drug administration into the NAc. In addition, the results revealed that intra-accumbal cannulation alone does not affect morphine-induced CPP (CS was 227.5 ± 35 in vehicle group and 219.6 ± 24.8 in vehicle + intra accumbal cannulation group).

**Fig. 2:**
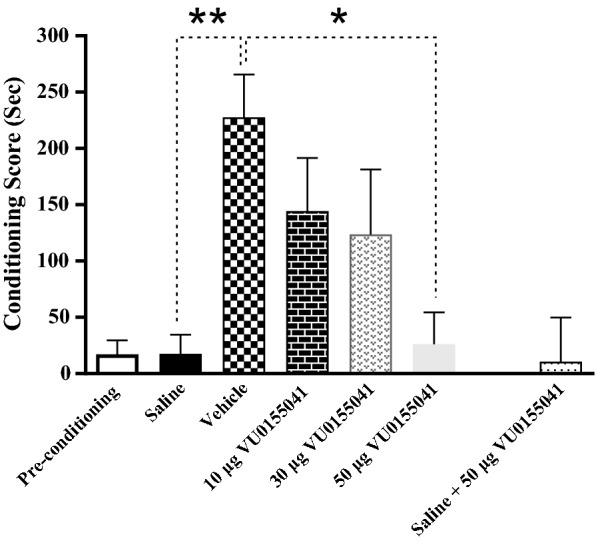
The effects of the administration of different doses of VU0155041, as a potent and selective mGluR4 agonist, (10, 30 and 50 μg/0.5 μL) into the NAc 5 min before the injection of morphine (5 mg/kg, sc) and administration of maximum dose of VU0155041 into the NAc region alone, on the conditioning days. Bars represent mean ± S.E.M (Pre-conditioning; n = 8, Saline; n = 6, Vehicle; n = 7, different doses of VU0155041 (10 μg/0.5 μL; n = 11, 30 μg/0.5 μL; n = 10 and 50 μg/0.5 μL; n = 8). *P < 0.05, **P < 0.01, Kruskal Wallis test followed by Dunn's multiple comparisons test

Shapiro–Wilk test was used to test the normality of the data. The Q–Q (quantile–quantile) plot for distribution of CS data is shown in Fig. 1. These data did not pass normality test, therefore the Kruskal Wallis test was used. The analysis the data revealed that there were substantial differences in the CS between the experimental groups (Fig. 2). The results also showed that there was a significant difference between the saline and vehicle group (*P* < 0.01). Simultaneous administration of intra-accumbal VU0155041 and systemic morphine during the acquisition phase reduced the rewarding properties of morphine in the CPP paradigm in a dose-dependent manner. (*P* < 0.05). The dose–response relationship is a central concept in toxicology and pharmacology [48]. The dose–response relationship describes the magnitude of the responses of an organism to a different dose of a chemical agent. Dose–response, which involves the principles of pharmacokinetics and pharmacodynamics, determines the required dose and frequency as well as the therapeutic index for a drug in a population. At low doses, there is a low-level effect increased with increasing dose; similarly, at high doses, there is a high-level effect that decreases with decreasing dose. Moreover, administration of the highest dose of VU0155041 (50 μg/0.5 μL) alone did not affect the CS in saline-treated animals (which received saline instead of morphine during the conditioning phase) (Fig. 2).

### Effects of intra-accumbal microinjection of mGluR4 receptor agonist, VU0155041, on the expression of morphine-induced CPP

**Fig. 3:**
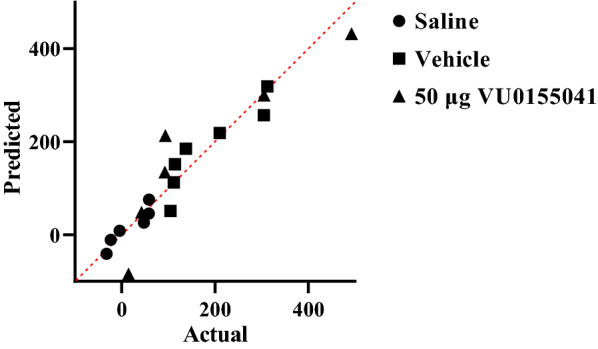
Q-Q plot for distribution of conditioning score data during post-conditioning (expression) phase**.** These data did not pass normality test**.** The data normality test was performed using Shapiro–Wilk test

**Fig. 4:**
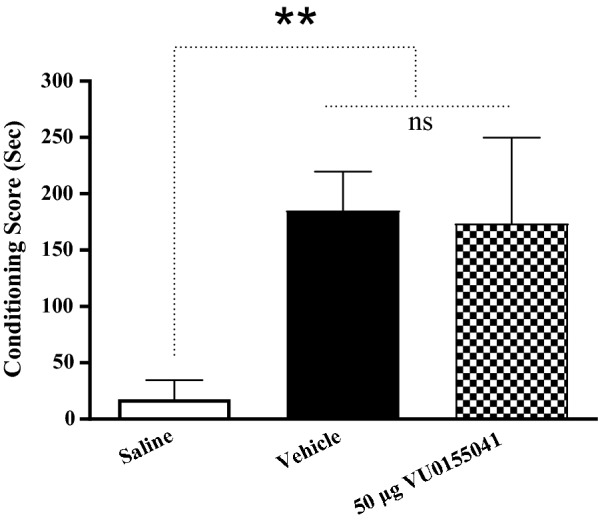
The effects of the administration of highest dose of VU0155041, as a potent and selective mGluR4 agonist, (50 μg/μL) into the NAc, 5 min before the test on the post-conditioning day. Bars represent mean ± S.E.M (Saline; n = 6, Vehicle; n = 7, VU0155041 (50 μg/side); n = 7). *ns* not significant, *p < 0.05. Kruskal Wallis test followed by Dunn's multiple comparisons test

Shapiro–Wilk test was used to test the normality of the data. The Q-Q plot for distribution of CS data during post-conditioning (expression) phase is shown in Fig. 3. These data did not pass normality test, so the Kruskal Wallis test was used. The analysis showed that intra-accumbal administration of VU0155041 (50 μg/μL) had no effect on the expression of morphine-induced CPP in morphine treated animals (*P* > 0.05, Fig. 4) compared with the vehicle group. It means that VU0155041 (50 μg/μL) could not reverse the morphine place preference.

### The effect of VU0155041 injection into the nucleus accumbens on motor activity of rats

**Fig. 5:**
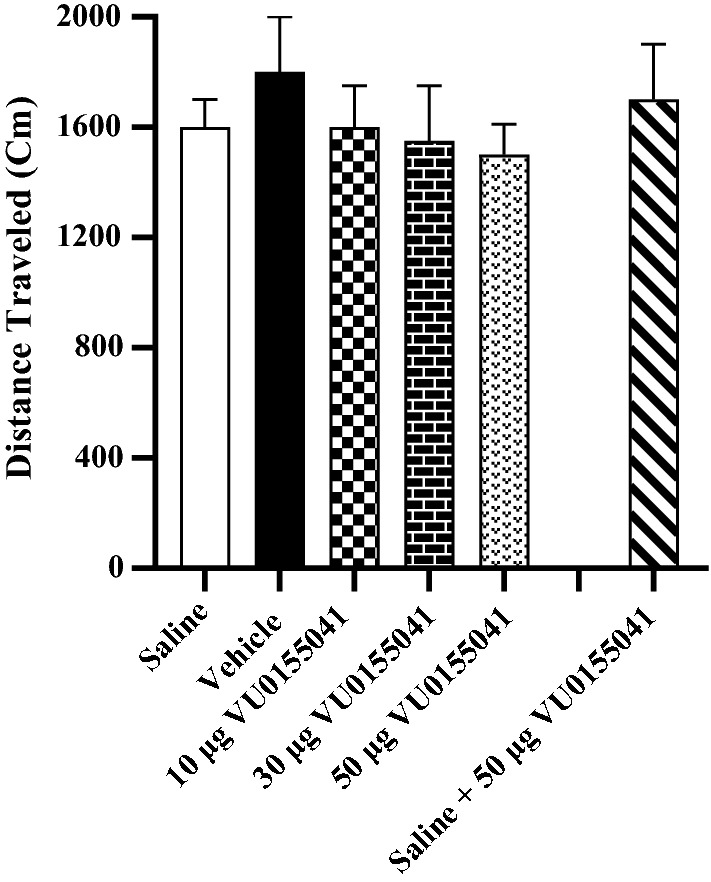
The effect of VU0155041 injection into the NAc on locomotor activity during morphine-induced CPP. Bars represent mean ± S.E.M (Saline; n = 7, Vehicle; n = 7, different doses of VU0155041 (10 μg/0.5 μL; n = 11, 30 μg/0.5 μL; n = 10 and 50 μg/0.5 μL; n = 8). One-way ANOVA followed by Newman-Keuls post-test

These data passed normality test, so the One-way ANOVA was used. One-way ANOVA followed by Newman-Keuls multiple comparison test [F [5, 45] = 0.3644, *P* = 0.8702] demonstrated that VU0155041 did not change the traveled distance during the 10 min test period (on the post-test day) in comparison with the vehicle and saline groups (Fig. 5).

## Discussion

In the present study, the effect of VU0155041 as a selective mGluR4 allosteric agonist within the NAc on development of morphine-induced CPP was investigated in rats. The main findings of the present study can be expressed as: (a) bilateral intra-accumbal microinjection of VU0155041 dose-dependently reduced the acquisition of morphine-induced CPP, (b) after conditioning, intra-accumbal activation of mGluR4 by VU0155041 at highest dose of 50 μg / 0.5 μL did not affect the expression of morphine-induced CPP in the rats, (c) administering the highest dose of VU0155041 into the NAc alone could not induce CPP, and (d) VU0155041 did not affect locomotor activity.

Previous studies have shown that mGluRs are involved in the acquisition and expression of morphine-induced CPP, mGluR2/3, [7] mGluR5 [22], mGluR7 [21], and mGluR8 [23] in NAc on acquisition and expression of morphine-induced conditioned place preference (CPP) in rats. This is the first study which has investigated the effects of intra-accumbal microinjection of mGluR4 agonist on the acquisition and expression of morphine-induced CPP.

Drugs have an additional level of selectivity (signaling selectivity or “functional selectivity”) beyond the traditional receptor selectivity [49].In the current study mGluR4 antagonist was not used and therefore it cannot be claimed that the observed effect is only due to stimulation of mGluR4. Moreover, even by using mGluR4 antagonist the observed effect could not be reversed since pharmacological antagonism may not show functional antagonism.

The NAc is a key brain region that receives and integrates convergent emotional, motivational, and reward-related signals that aid in regulating behavioral output [50]. These signals are thought to be mediated, in part, by glutamatergic inputs from several brain regions including the VTA, basolateral amygdala, medial prefrontal cortex, and the ventral hippocampus [51, 52]. Excitatory afferents to the NAc are thought to facilitate reward seeking by encoding reward-associated cues. Recent optogenetic studies, for example, have revealed that activation of glutamatergic inputs from the amygdala or the ventral hippocampus to NAc facilitates reward seeking [52, 53]. On the other hand, it has been reported that morphine eliminates the inhibitory effects of dopamine on glutamatergic inputs on NAc neurons and enhances glutamate activity on this nucleus [54]. The mGluR4 is primarily localized presynaptically in glutamatergic terminals [45] and acts as inhibitory presynaptic receptor and reduces synaptic transmission [46]. Based on the results presented in this study, it can be concluded that VU0155041 blocks the rewarding properties of morphine by reducing glutamate release form glutamatergic inputs to the NAc. Interestingly, Barrett et al. (2012) suggest that the specific pathway releasing glutamate is not as important as the amount of glutamate that is released [52]. It cannot be completely excluded that the observed effect of VU0155041 injections on acquisition of morphine CPP is specific to intra-accumbal infusion of the drug rather than diffusion/action of at other brain regions.

Studies on mGluR4 have shown that mGluR4 is involved in locomotor activity and regulation of motor stimulation induced by intraperitoneal (i.p.) injection and oral administration of ethanol [39]. In the current study the effects of this drug injection in open field test were checked and no significant effect was found (data not presented). In addition, the tracking system could record animal locomotion during all session (pre-conditioning, conditioning and post-conditioning phases of the CPP paradigm) and also no significant effect on traveled distance and velocity was found which means that this agent had no effect on locomotor activity and exploring behavior. Of course, learning and memory is engaged in classical conditioning such as CPP paradigm and drug may influence learning and memory [55].

Recent studies have identified mGluRs as potential targets for the treatment of drug addiction. There is type specificity and phase dependency for the role of mGluRs in morphine induced-CPP [7, 21, 22, 37, 38]. Despite the importance of glutamate in drug dependence, only a few studies have demonstrated anti-addictive activity in the group III of mGluRs (mGluR4, mGluR6, mGluR7, and mGluR8).

The NAc include two main parts: core and shell. The NAc core is responsible for the evaluation of reward and initializing reward-related motor action [56–58]. Hence, The NAc core is essential for acquiring drug-taking behaviors and cue-elicited drug-seeking responses. For psychostimulant drugs, learning drug reward associations is largely dependent on dopaminergic and glutamatergic signaling within the NAc core, whereas reinstatement is mostly driven by glutamate [59, 60]. In the current study the selective mGluR4 allosteric agonist (VU0155041) was injected into the NAc and because of the injection site, VU0155041 may have affected both parts of the NAc and therefore the role of each part cannot be excluded. However, the inhibitory effects of mGluR4 activation on morphine induced CPP acquisition can be mostly related to these receptors in NAc core which remained to be elucidated.

Group III mGluRs are generally located presynaptically and regulate neurotransmitter release and activation of these receptors cause glutamate release inhibition [61]. mGluR4 receptors have been identified as attractive targets for treating anxiety disorders [62]. In addition, Davis and colleagues in 2012 have shown that mice lacking or deficient in mGluR4 were associated with increased anxiety [63]. Their data suggest that pharmacological activation of the mGluR4 may be useful in reducing anxiety alterations in fear learning mechanisms likely participate in the development and/or maintenance of anxiety disorders. Anxiety disorders and substances use disorders often occur together, but the strength of this association and their apparent order of onset differ across studies [64, 65]. Morphine dependent animals' have been shown to have enhanced anxiety levels [66]. Due to the fact that rats’ performance in CPP apparatus is related to the learning and anxiety mechanisms [67], some effects of mGluR4 activation in the NAc on acquisition of morphine induced CPP may be related to the role of this receptor on anxiety state.

The present study revealed that after conditioning, intra-accumbal activation of mGluR4 by VU0155041 did not affect the expression of morphine-induced CPP in the rats. In contrast to the observations, Zaniewska and colleagues have shown that administration of either mGluR4 orthosteric agonist LSP1-2111 or a positive allosteric modulator of mGluR4 Lu AF21934 attenuated the expression of cocaine sensitization [40]. One explanation for the difference in the effects of mGluR4 receptor agonists on the behavioral responses to cocaine and morphine could be that these two substances have different neuropharmacological mechanisms and neuroanatomical sites of action. Moreover, this incongruity could be explained by the animal species used, behavioural model of substance abuse, and/or the type and dose of agonists.

## Conclusion

Although the role of mGluR4 in reward is well known, more experiments are needed to conclude that VU0155041 blocks the rewarding properties of morphine. In conclusion, the data of the present study extend present knowledge about the effects of pharmacological stimulation of mGluR4 on the behavioral responses to morphine in rats and demonstrate that activation of mGluR4 in NAc confers an inhibitory effect on the acquisition of morphine induced CPP while it had no effect on the expression of morphine-induced CPP. Future studies are needed to characterize the specific action mechanisms of mGluR4 in acquisition and expression of morphine-induced place preference in rats.

## Materials and methods

### Ethics statement

All experimental procedures using rats were conducted in accordance with the animal care and use guidelines approved by the institutional ethics committee at Hamadan University of Medical Sciences (Ethic code: IR.UMSHA.REC.1397.784) and were performed in accordance with the National Institutes of Health Guide for Care and Use of Laboratory Animals. All efforts were made to minimize suffering. The operations that could cause pain and distress were performed in another room in the absence of other animals.

### Animal

Male Wistar rats (200–250 gbodyweight) were purchased from animal breeding colony of Hamadan University of Medical Sciences. They were maintained on cycle with 12 h of light and 12 h of darkness each day (light on at 7 AM) and had access to freely available food and water in their home cages. Ambient temperature (22 °C ± 2 °C) was kept constant.

### Drugs

In the present work the following drugs were used: Morphine sulfate (Temad, Iran) was dissolved in normal saline (0.9% NaCl); cis-2-[[(3,5-Dichlorophenyl)amino]carbonyl]cyclohexanecarboxylic acid (VU0155041) (Tocris, UK), a selective mGluR4 allosteric agonist was also dissolved in normal saline (0.9% NaCl). VU0155041 is a mixed allosteric agonist/positive allosteric modulator (PAM) of mGluR4 [68]. VU0155041 is approximately eightfold more potent than PHCCC (N-Phenyl-7-(hydroxyimino) cyclopropa[b]chromen-1a-carboxamide) and does not show any significant potentiator or antagonist activity at other mGluR subtypes. It is soluble in an aqueous vehicle. It also enhances the activity of glutamate about eightfold [68]. In this study, the saline group animals received subcutaneous injection of saline (as a solvent of morphine) instead of morphine during the conditioning phase (1 ml/kg; s.c.; n = 6/group). The vehicle group animals received subcutaneous injection of morphine (5 mg/kg; s.c.; n = 7) during the conditioning phase along with intra-accumbal microinjection of saline (instead of VU0155041).

### Stereotaxic surgery and drug administration

The rats were anesthetized by Ketamine/Xylazine combination (K, 100 mg/kg; X, 10 mg/kg) and placed in the stereotaxic device (Stoelting, USA) with the incisor bar set at approximately 3.3 mm below horizontal zero in order to achieve a flat skull position. Next, an incision was made to expose the rats' skull and two points were determined and drilled into the skull at stereotaxic coordinates of 1.45 ± 0.3 mm anterior to bregma, ± 1.5 mm lateral to the sagital suture. Two guide cannulae (23-Gauge) with 12 mm length were inserted into the holes aiming at the NAc, 6.5 mm down from top of the skull according to the atlas of rat brain (Paxinos and Watson, 2007). The guiding cannulae were anchored with a jeweler's screw and the incision was closed with dental cement. After surgery, the dummy inner cannulae that extended 0.5 mm beyond the guiding cannulae were inserted into the guiding cannulae and left in place until injections were made. All rats were allowed to recover for one week before starting the behavioral testing.

### Intra-accumbal injection

The rats were gently restrained by hand and the dummy cannulae were removed from the guiding cannulae. Drugs were directly injected into the NAc through the guiding cannulae using injector cannulae (30-gauge, 1 mm below the tip of the guiding cannula). Polyethylene tubing (PE-20) was used for attaching the injector cannula to the 1-μl Hamilton syringe. Selective mGluR4 allosteric agonist, VU0155041, was administered into the NAc at different doses (10 μg/0.5 μL saline (n = 11), 30 μg/0.5 μL saline (n = 10) and 50 μg/0.5 μL saline (n = 8) per side) [69, 70]. The volume of drug or saline injection into NAc for all groups was 0.5 μl per side. Bilateral injections were performed over a 50 s period and the injection cannulae were left in the guiding cannulae for an additional 60 s in order to facilitate the drug delivery.

### Place conditioning apparatus and protocol

A three-chamber CPP apparatus was used. The CPP apparatus was divided into two equal-sized side chambers (30 × 30 × 40 cm) and one middle chamber (30× 15 × 40 cm) being the null section which connected the two side chambers. Both chambers had white backgrounds with black stripes in different orientations (vertical vs. horizontal). To provide a tactile difference between the chambers, one of them had a smooth floor, while the other chambers had a net-like floor. The CPP protocol has been previously described [21, 23] and an unbiased allocation was used. Rats with a neutral preference (45–55% for either side) were randomly allocated their drug-paired side (unbiased allocation). In the CPP paradigm, the conditioning score (CS) and distance traveled were calculated based on a video recorded by a CCD camera with 30 frames per second (30 fps) resolution. The camera was placed 2 m above the CPP boxes and the locomotion tracking was measured by Maze Router homemade software, a video tracking system for automation of behavioral experiments. The CPP paradigm was held for 5 consecutive days, which consisted of three distinct phases: pre-conditioning, conditioning and post-conditioning [21, 22].

### Pre-conditioning phase

On day 1, each rat had free access to all chambers of the device for 10 min. Animal movements were recorded by Maze Router tracking software and analyzed on the same day. Three rats with any chamber preference were omitted from the study. The rats were randomly assigned to one of the two groups (odd and even) for place conditioning [21].

### Conditioning phase

The dose–response for morphine on conditioned place was evaluated in the CPP paradigm [7, 21, 22]. Different doses of morphine sulfate were injected into animals (0.25, 0.5, 0.75, 1, 2.5, 5, and 10 mg/kg; s.c.). Compared with animals receiving subcutaneous saline injection, significant increase in CPP score was observed at the doses of 5 and 10 mg/kg. It was revealed that 5 mg/kg of morphine is lowest effective dose. On days 2, 3 and 4, the conditioning phase of morphine (also known as the acquisition phase) was performed. Each group of animals was randomly divided into even or odd. Odd animals received subcutaneous (SC) injection of saline and morphine (5 mg/kg) pairing in alternative morning and afternoon design with an interval of 6 h. The vice versa program for even animals was done. This phase consisted of a 3-day schedule of conditioning sessions. A total of six sessions (30 min each) were performed. During these 3 conditioning days of 3 sessions, the animals under the drug influence were confined to one chamber. During the other three sessions, they were injected with saline while confined to the other chamber. Access to the other chambers was blocked on these days. Place preference was calculated as a preference score (time spent in drug paired zone − time spent in the saline paired zone) [21, 22]. During this phase, saline group animals received saline in both chambers during alternative morning and afternoon design with an interval of 6 h. Locomotor data were also collected throughout CPP testing in order to assess the development of behavioral sensitization.

### Post-conditioning phase

On day 5, the partition was removed and the rats could access the entire device. The mean time spent for each rat in both chambers during a 10-min period was recorded. In order to calculate the conditioning score, the difference in the time spent for the drug- and saline-paired places was considered as the preference criteria. In the acquisition tests, no injection was given on the post-conditioning day.

## Experimental design

### The effect of intra-accumbal administration of mGluR4 allosteric agonist (VU0155041) on the acquisition of morphine-induced CPP

To investigate the effects of mGluR4 agonist on the acquisition of morphine-induced CPP, bilateral intra-accumbal injection of VU0155041 (10, 30 and 50 μg/0.5 μL) [71] was done 5 min prior to each morphine injection during the conditioning phase (once daily for 3 days). During this phase, vehicle group animals received saline (0.5 μL) instead of VU0155041 in the NAc prior to SC injection of morphine (5 mg/kg; SC). Moreover, to rule out the possibility that VU0155041 administration alone had rewarding or aversive effects on the CPP, a separate group of rats received the highest dose (50 μg/0.5 μL) of VU0155041 prior to saline injection (1 mL/kg; SC) instead of morphine during the conditioning days.

### The effects of intra-accumbal VU0155041 injection on the expression of morphine-induced CPP

In order to examine the effects of the highest dose of VU0155041 (50 μg/ 0.5 μL saline, n = 6) on the expression of morphine-induced CPP, the rats were bilaterally given VU0155041 in the NAc 5 min prior to CPP test. In addition, vehicle group animals (n = 7) received saline (0.5 μL) through the NAc instead of VU0155041 before CPP test in post-conditioning phase. Animals in the saline group received saline instead of morphine during the conditioning phase (n = 6).

### Locomotor activity measurement

The locomotor activity of each rat was recorded using the locomotion tracking apparatus by a Maze Router tracking software. In these experiments, the total distance traveled (in centimeters) by each rat was measured in pre- and post-tests for all groups.

### Histology

**Fig. 6:**
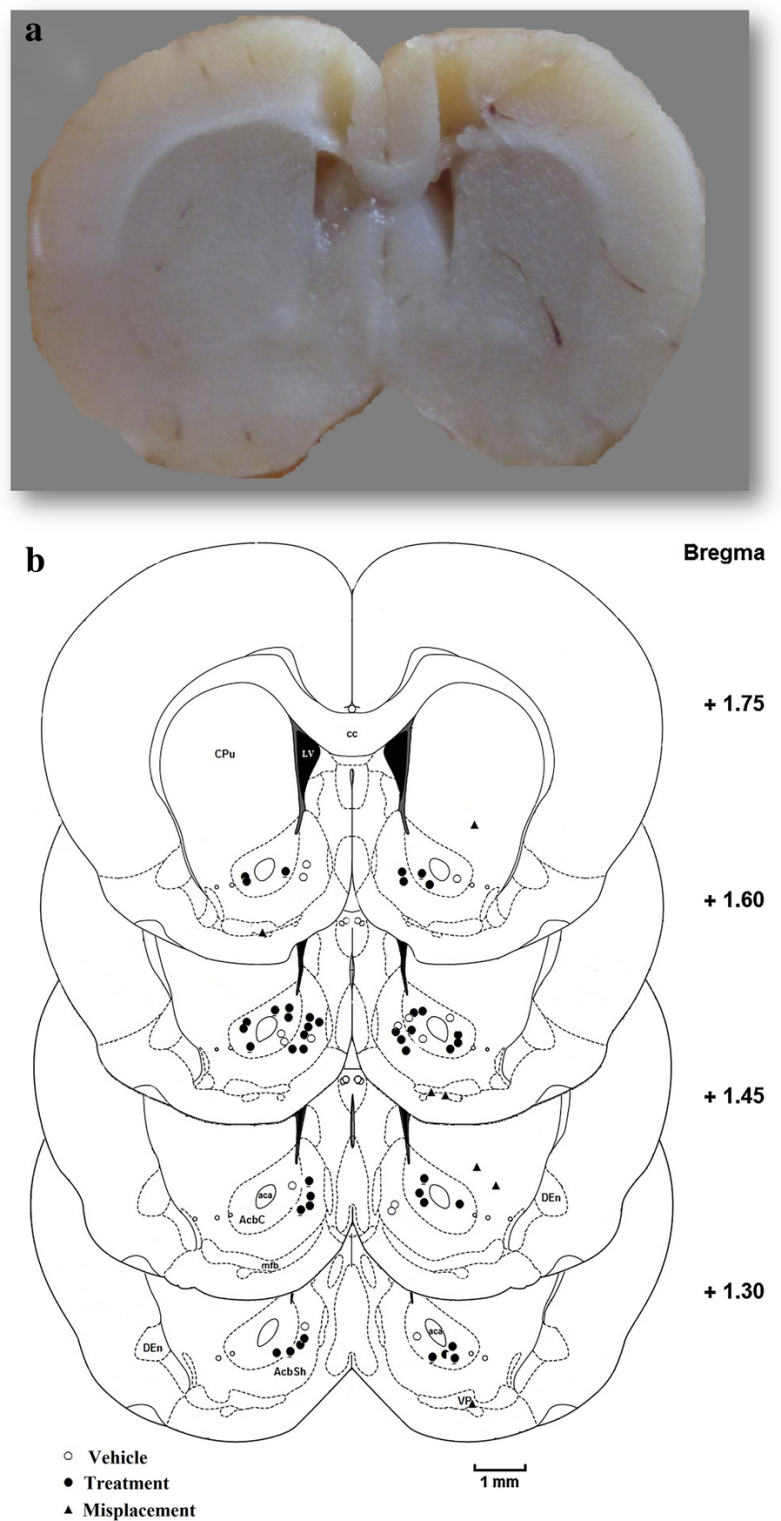
Histology.** a** Coronal photomicrograph of the bilateral microinjection sites in the nucleus accumbens (NAc) and **b** schematic illustration of rat brain coronal sections showing the approximate location of the NAc injection sites. The numbers indicate the anterior–posterior coordinates relative to bregma. Atlas plates were adapted from Paxinos and Watson (Paxinos and Watson, 2007). aca, anterior commissure, anterior part; CPu, caudate putamen (striatum); gcc, genu of the corpus callosum; NAc, nucleus accumbens; scale is 1 mm. Both Fig. 1a and b shown are the authors' work. (Vehicle; n = 14 Treatment; n = 43, Misplacement; n = 7)

After the behavioral tests and data collection, the rats were anesthetized with Ketamine and Xylazine and then sacrificed in order to check for proper placement of cannulae in brain areas. Ketamine–xylazine is a commonly used combination for anesthesia and euthanasia in rat [72] and mice [73]. In the present work, the rats were anesthetized (by combination of Ketamine 300–360 mg/kg + xylazine 30–40 mg/kg; i.p.) and sacrificed by decapitation. Next the brains were removed, and fixed in 10% formalin solution. 50 µm coronal sections of the brain tissue were cut using a rotatory microtome. The correct placement of cannulae was investigated using rat brain atlas. Only the rat brains with correct cannulae placement (Fig. 6a, b) were chosen for final data analysis. Animals with cannula misplacement (n = 7) were excluded from the study.

### Statistics

The data were processed by existing commercial software GraphPad Prism® 8.0.2. Shapiro–Wilk test was used for data normality test. If the data passed normality test (Shapiro–Wilk test greater than 0.05), the one-way analysis of variance (ANOVA) followed by post hoc analysis (Newman–Keuls multiple comparison test) was used. But If the data did not pass normality test (Shapiro–Wilk test less than 0.05), Kruskal Wallis test was used followed by Dunn's multiple comparisons test. The Kruskal Wallis test is the non parametric alternative to the one-way ANOVA. Multiple student's *t*-test was used to compare pre-conditioning with saline or highest dose of VU0155041 (50 μg/0.5 μL). *P*-values less than 0.05 (*P* < 0.05) were considered to be statistically significant [21, 22].

## Data Availability

The data are available for any scientific use with kind permission.
